# Long-term annual trends in suicide rates and characteristics of suicide cases in prisons in Japan

**DOI:** 10.3389/fpubh.2025.1732499

**Published:** 2026-01-07

**Authors:** Naohiro Yonemoto, Yoshitaka Kawashima

**Affiliations:** 1 Department of Biostatistics, Faculty of Medicine, University of Toyama, Toyama, Japan; 2 Department of Public Health, Juntendo University School of Medicine, Tokyo, Japan; 3 Clinical Psychology Course, Department of Psycho-Social Studies, School of Arts and Letters, Meiji University, Tokyo, Japan

**Keywords:** Japan, prison, suicide, suicide rate, trends

## Abstract

This study aims to investigate suicide mortality rates and their time trends in prisons against the national level and related facilities, and to identify characteristics of suicide cases in Japan. The study design of this study was a nationwide cross-sectional study. It targeted prisoners and individuals incarcerated in all facilities, such as prisons, under the control of Ministry of Justice in Japan. The suicide rate (per 100,000) was calculated, and its annual trend described. The number and percentage of individuals by each measure were described in suicide cases. The suicide rate from 1952 to 2022 showed considerable annual variation (minimum 5.5 to maximum 46.8). No clear parallel trend was visually observed with the year trends in suicide rates for the Japanese population, male, or female. There were 184 suicides from 2010 to 2020. The sex was 89% male and 10% female. The age distribution showed the largest groups were those in their 30 s (26%) and 40 s (34%). The suicide rate in Japan was not found to be as high as in other developed countries. The trends in annual suicide rates differed significantly, and an unclear correlation was observed with the suicide rate in all of Japan. Suicides among middle-aged men were high. The information on suicide and for prevention was so limited, and further research is needed.

## Introduction

Suicide is a major global public health concern. It also affects prisoners and even incarcerated individuals. Research indicated that suicide rates among prisoners exceed those in the general population, highlighting the need for targeted prevention strategies within correctional facilities (1, 2). A meta-analysis reported prison suicide rates ranging from 24 to 89 per 100,000 person-years (3). However, reporting on prison suicide rates at the national level remains limited even in developed countries, particularly in Asian nations (4).

In Japan, demographic shifts are occurring among prison populations (Ministry of Justice in Japan, 2021), with increasing proportions of older inmates and those with mental disorders—both high-risk groups for suicide. Japan has one of the highest suicide rates in global (5). However, the epidemiology of suicide in these facilities in Japan remains understudied. Understanding annual trends of suicide and characteristics of prison suicides in Japan is crucial for developing targeted prevention strategies and policy interventions.

This study aims to investigate suicide mortality rates and their time trends in prisons and related facilities, and to identify characteristics of suicide cases in Japan. The research hypothesis is that while suicide rates in prisons and related facilities in Japan are higher than the national suicide rate, the trends do not follow the same pattern as previous studies (1, 2).

## Methods

The study design of this study was a nationwide cross-sectional study. It targeted prisoners and individuals incarcerated in all facilities, such as prisons, under the control of Ministry of Justice in Japan.

### Measures

The number of suicides (the numerator of the suicide rate) and the total number of inmates across all facilities (the daily average inmate population, serving as the denominator) were obtained from the Statistics on Correction (1952–2022), official statistical reports administered by Ministry of Justice in Japan (6). Regarding the characteristics of suicide cases, data from CrimeInfo (crimeinfo.jp) on “report of accident occurrence in prisons” (2010–2022) was used (7) CrimInfo is a non-profit organization that provides information on the death penalty system and issues within prisons. The data in CrimInfo were obtained from the public vital statistics, reports disclosed after requests to the Ministry of Justice in Japan, and data from newspapers in Japanese. We only used the information as background for suicide cases from the data disclosed in CrimInfo.

Measures characterizing suicide cases included gender, age (10-year age groups), attributes of people (prisoner, defendant, others, unknown), facility where death occurred (prison, jail and detection center, juvenile detention center, other), year period (2010–2014, 2015–2019, 2020–2022), and month of occurrence (January–March, April–June, July–September, October–December). Definitions of measures conformed to the statistics on correction in Japan.

The annual suicide rates for Japan as a whole and by gender were derived from demographic statistics and data from the yearly report for suicide prevention by ministry of health, welfare and labor (8, 9).

### Statistical analysis

The suicide rate (per 100,000) was calculated, and its annual trends described. For supplemental references, the suicide rates for the Japanese population as a whole and by sex were also represented. The number and percentage of individuals by each measure were described in suicide cases.

## Results

### Year trends

**Figure 1:**
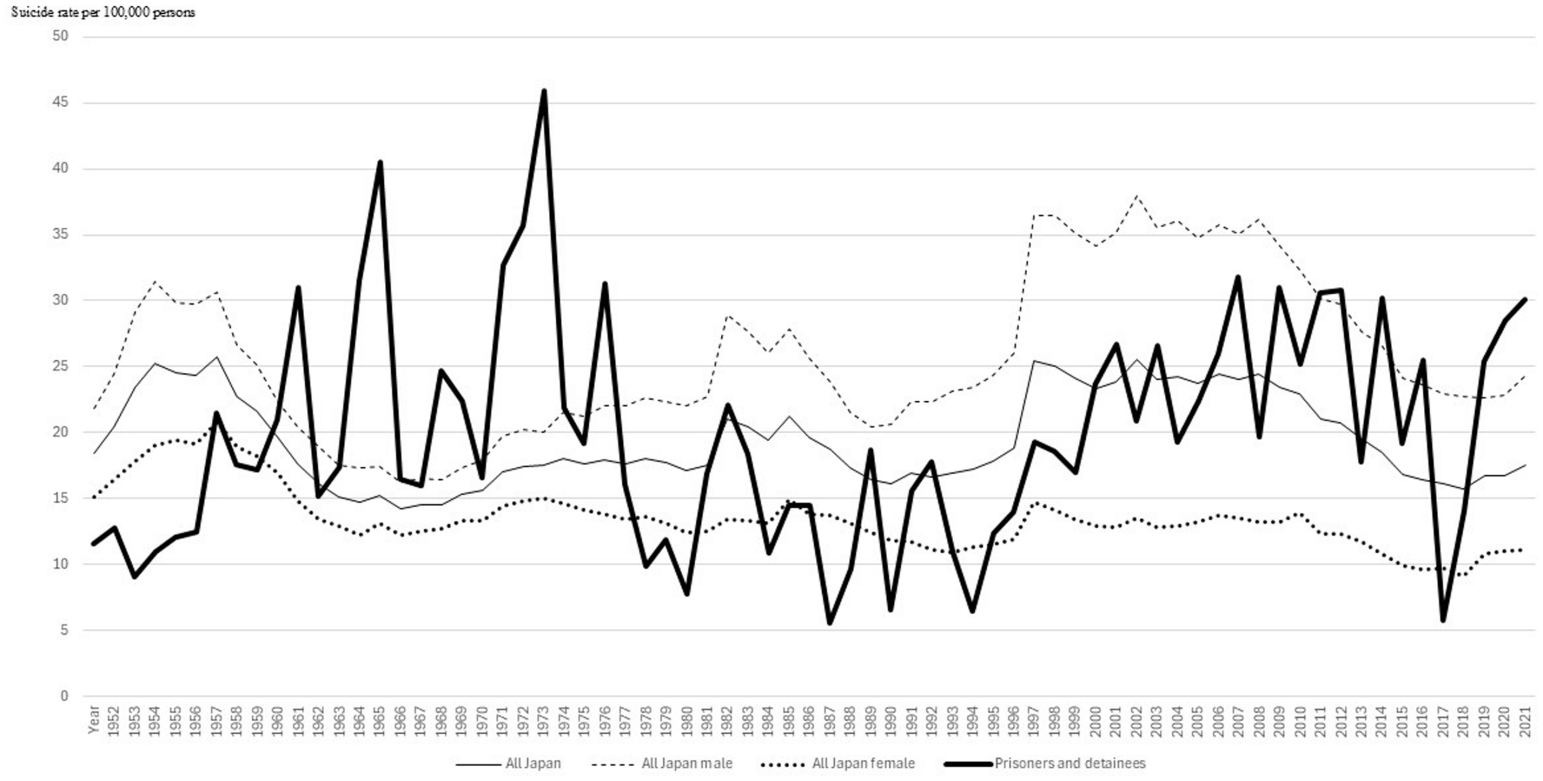
Long-term annual trends in suicide rates in prisons in Japan (1952–2022).

The suicide rate showed considerable annual variation (minimum 5.5 to maximum 46.8), from 1952 to 2022 (Figure 1). From 2010 to 2022, there was a range from a minimum of 5.9 to a maximum of 31.8, from 2010 to 2022. No clear parallel trend was visually observed with the annual trends in suicide rates for the Japanese population, male, or female.

### Characteristics of suicide cases

There were 184 suicides from 2010 to 2022 (Table 1). The sex was 89% male and 10% female. The age distribution showed the largest groups were those in their 30 s (26%) and 40 s (34%). Prisoners were 60% and defendants for 37%. The distribution of place of the cases was similar. There were about half cases in 2015–2019 as in 2010–2014.

**Table 1:** Characteristics of suicide cases in prison from 2010 to 2022 (*n* = 184).

Characteristics		*N* (%)
Sex	Male	164 (89.1)
	Female	19 (10.3)
	Unknown	1 (0.5)
Age	20–29	16 (8.7)
	30–39	48 (26.1)
	40–49	63 (34.2)
	50–59	22 (12.0)
	60–69	23 (12.5)
	70–79	9 (4.9)
	80	1 (0.5)
	Unknown	2 (1.1)
Type	Prisoner	111 (60.3)
	Defendant	68 (37.0)
	Other	4 (2.2)
	Unknown	1 (0.5)
Place of the case	Prison	107 (58.2)
	Jail and detection center	63 (34.2)
	Juvenile detention center	7 (3.8)
	Other	7 (3.8)
Years	2010–2014	93 (50.5)
	2015–2019	53 (28.8)
	2020–2022	38 (20.7)
Months	1–3	51 (27.7)
	4–6	51 (27.7)
	7–9	43 (23.4)
	10–12	39 (21.2)

## Discussion

This study revealed the annual trends and the characteristics of those who died by suicide in suicides in prisons and related facilities. The study presented that no clear parallel trend was visually observed with the year trends in suicide rates for the Japanese population, male, or female. This study presented the overall and time trends findings at a descriptive level. But analytical insights and the relationships between risk groups and factors, such as age group, remain subjects for future research. The suicide rate in Japan was not found to be as high as in other developed countries. Because a systematic review reported that the annual suicide rate per 100,000 was from 10 to 180 in Europe, Australia, and North America (1). Also, another systematic review reported that the median in suicide incidence was 80 (interquartile 51–102) in high-income countries (2). While people in these facilities constitute a special group and environment, making comparisons difficult, the suicide rate within these institutions fluctuated, being higher in some years and lower in others compared to suicide rates in the general population in Japan. The trends in annual suicide rates differed significantly, and no correlation was observed with the suicide rate in all of Japan.

Most suicide cases were among males. This is likely due to the higher proportion of males in these facilities. It might be because the suicide rate among men is higher than that among women in the Japanese population. The number of suicides among middle-aged individuals was high. These patterns might be different from those observed in the general Japanese population, because the population in prison would be a special one. The case count in prison appears roughly halved in 2015–2019 compared with 2010–2014. The reason is unclear, but the suicide rate in the general population in Japan was declining between the periods. This may be related to that. While the composition of the target population is unclear, this finding may be significant. In the closed environment of a prison, factors such as disconnection from society, loss of social status, distance from family and external supports, unstable circumstances, psychological shock from incarceration, and uncertainty about the future would be thought to increase suicide risk.

This study has several limitations. The first is the issue of data sources. This data examined suicide based on statistics concerning causes of death. Causes of death were ultimately based on reports from the Ministry of Justice and differ from national mortality statistics. Therefore, there were likely potential for misclassification or underreporting in cases of death from illness or other causes. There were also unknowns regarding the characteristics of suicide cases, and the quality of the information had limitations. The second limitation concerned the denominator information for suicide rates. This study used the average number of people in all prisons and related facilities, but this denominator may be too broad. The suicide rate could change depending on what is used as the denominator. Third is the scarcity and lack of transparency in background information. There was no information on the circumstances of suicide, the method of suicide, or the case with psychiatric history, substance dependence, physical medical history, or history of suicidal behavior. This severely limits the information available for considering suicide prevention. Therefore, we believe it is necessary to collect information on risk factors as reported in a previous review (10) and to better understand the actual situation, and to consider prevention strategies. The fourth issue was the difficulty of comparison. Criminal justice systems vary significantly between countries, even developed countries. Therefore, it might be difficult to compare the same population. In Japan, persons under suspicion may be detained at police stations before arrest, but this study did not include that population of the scope. In addition, prison data may exhibit exaggerated annual fluctuations due to small numerators and denominators. Consequently, increases or decreases depicted on the graph of suicide rate may be overestimated, necessitating caution in interpreting such variations. These might be because of three factors: (1) the population in prison, which forms the denominator for suicide rates, is not large relative to the general population; and (2) the small number of observed suicides, which can lead to random fluctuations having a disproportionate impact on calculated rates. Furthermore, (3) factors such as concentrated occurrences in specific institutions during years may contribute to variations in annual rates.

## Conclusion

This study revealed the annual trends and the characteristics of those who died by suicide in suicides in prisons and related facilities. The suicide rate in Japan was not found to be as high as in other developed countries. The trends in annual suicide rates differed significantly, and no correlation was observed with the suicide rate in all of Japan. Suicides among middle-aged men were high. The information on suicide and for prevention was still limited, and further research is needed.

## Data Availability

The original contributions presented in the study are included in the article/supplementary material, further inquiries can be directed to the corresponding author.
